# Psilocybin as a Treatment for Psychiatric Illness: A Meta-Analysis

**DOI:** 10.7759/cureus.31796

**Published:** 2022-11-22

**Authors:** Ricardo Irizarry, Amelia Winczura, Omar Dimassi, Navpreet Dhillon, Annu Minhas, Jeanpaul Larice

**Affiliations:** 1 Behavioral Health, South Texas Health System, Edinburg, USA; 2 Psychiatry and Behavioral Sciences, Tropical Texas Behavioral Health, Edinburg, USA; 3 Psychiatry and Behavioral Sciences, Saint James School of Medicine, Chicago, USA

**Keywords:** meta-analysis, magic mushrooms, psychedelics, anxiety, depression, psychiatric illness, psilocybin

## Abstract

Psilocybin is an emerging potential therapy for the treatment of psychiatric illnesses. Microdosing has been shown to result in an overall improvement in patients with anxiety, depression, obsessive-compulsive disorder, post-traumatic stress disorder, and substance abuse. This meta-analysis explores and compiles prior research to make further inferences regarding psilocybin and its use for the treatment of psychiatric illness along with its safety and efficacy.

Database searches were conducted to identify peer-reviewed randomized controlled trials and clinical trials mentioning psilocybin use and psychiatric illness. A Preferred Reporting Items for Systematic Reviews and Meta-Analyses flow diagram was created and analysis was run on the nine articles that met all established inclusion criteria. An event is defined as a participant who showed improvement, in a quantitative method, from baseline after the use of psilocybin. Another analysis was done using depression severity (Quick Inventory of Depressive Symptomatology 16-Item Self Report, QIDS-SR16) at baseline and after the use of psilocybin.

Analyses of the original data and the nine articles showed a great deal of heterogeneity with an I^2^ value of 73.68%, suggesting that the studies in this meta-analysis cannot be considered to be studies of the same population. The Q value of 30.4 was higher than 15.507, which is the critical value for eight degrees of freedom found in a chi-square distribution. This Q value showed a high degree of variation and lacked significance. The second meta-run on QIDS-SR16 scores from three studies showed a Q value of 1.16 which was lower than 5.991, the critical value for two degrees of freedom found in a chi-square distribution. The I^2^ statistic for this second meta-analysis was -73% which can be equated to zero. This indicated that the data were homogeneous or that there was no observed heterogeneity. Due to low heterogeneity, the fixed-effects model was used. Based on this meta-analysis, psilocybin seems to show symptom improvement in some psychiatric illnesses. The effectiveness of psilocybin microdosing and the use of psilocybin, in general, need to be studied further to determine the efficacy and safety of potential applications in psychiatry.

## Introduction and background

Nearly 50 million adults or 19.86% of the United States (US) population are affected by some form of a psychiatric or mental health illness. Roughly one in five adults in the US experiences mental illness each year [1]. The prevalence of mental illness among US adults, within the general population, in descending order, is generalized anxiety disorder (GAD) at 19.1%, major depressive disorder (MDD) at 8.4%, post-traumatic stress disorder (PTSD) at 3.6%, bipolar disorder at 2.8%, and obsessive-compulsive disorder (OCD) at 1.2% [1]. Treatment-resistant psychiatric disorders unresponsive to available standard therapies have been on the rise. It is estimated that roughly 20-60% of all patients with psychiatric illnesses are treatment-resistant [2].

Psychedelics have been explored as an alternative treatment, particularly for treatment-resistant psychiatric illnesses. Psychedelics are also known as hallucinogens and are a class of drugs that cause changes in cognitive processes, mood, and perception of reality. The most common hallucinogens are lysergic acid diethylamide (LSD), dimethyltryptamine (DMT), psilocybin, mescaline, and 251-NBOMe [3]. Although past research has been inconsistent, there has been a renewed interest in studying the potential beneficial effects of psilocybin. Psilocybin was studied extensively when it was originally discovered. In the 1960s, research on the fungus stopped due to growing misconceptions stemming from its widespread recreational use, with users experiencing intense and often overwhelming effects following large doses. In subsequent studies, however, researchers showed that individuals respond differently when taking smaller doses and that these smaller doses may indeed have medical benefits [4]. Researchers showing psilocybin has potential beneficial effects in small doses in treating psychiatric illnesses has led to its resurgence in research. While psilocybin is not currently an approved treatment for any medical condition, there is promising evidence showing its potential benefits.

Psilocybin, also called magic mushrooms, is a schedule one controlled substance. Psilocybin mushrooms are primarily found in South America, Mexico, and subtropical regions of the US, and are ingested either via brewing tea or consuming the mushrooms raw or dried [5]. Psilocybin mushrooms have long, light-colored, slender stems and a dark underside where the gills are; the cap is dark brown around the edges and light brown or white toward the center. When dried, the fungus is usually an off-white to rusty brown color [1]. The chemical formula for psilocybin is 4-phosphoryloxy-N,N-dimethyltryptamine. This chemical formula was first isolated by Swiss chemist Albert Hoffman in 1957. Hoffman had extensive experience researching psychedelics, and he was also the first to isolate LSD in 1938 [6]. Psilocybin interacts primarily with serotonin receptors, specifically 5-HT1A, 5-HT1D, 5-HT2A, and 5-HT2C receptor subtypes [7]. Psilocybin is predominantly metabolized by the liver and the following metabolites are formed: 4-hydroxy-N,N-dimethyltryptamine (psilocin); d 4-hydroxyindole-3-yl-acetaldehyde (4H1A); d 4-hydroxyindole-3-yl-acetic-acid (41-IIAA); and d 4-hydroxytryptophol (41-IT) [7]. After oral ingestion, psilocybin is dephosphorylated to generate the phenol compound psilocin. This facilitates crossing the blood-brain barrier and produces serotonergic effects. Psilocin is usually detectable in the plasma within 20-40 minutes [7,8].

Psilocybin is eliminated through the kidneys, where two-thirds of the excretion usually occurs within three hours of ingestion [7]. Once psilocybin has crossed the blood-brain barrier and is bound to serotonergic receptors, it causes an increase in neurotransmitter activity. Specifically, it causes an increase in intracellular signaling cascades in pyramidal cortical neurons modulating downstream signaling proteins such as early growth response proteins 1 (ERG1) and ERG2 [9]. These downstream signaling proteins are zinc finger proteins whose induction has been shown to cause neuronal activity and has been thought to increase neuronal plasticity, strengthening the synapse over time. In non-human clinical trials, psychedelic compounds have been shown to promote neuroplasticity by increasing the growth and complexity of neurons, allowing for increased learning [10].

The physical and emotional effects of psilocybin include euphoria, hallucinations, dissociation, an increase in blood pressure, and an altered state of cognition. Some researchers have shown through studies to have subacute effects on empathy, creative thinking, and subjective well-being. These aspects of self-identity, perception, and other existential traits or outlooks are greatly influenced by psychedelics. Traits including ego-dissolution, as well as enhancement of outlooks such as a positive sense of self, overall quality of life, and meaningful existence, are common themes and examples of some of the positive subjective effects reported in patients receiving psilocybin experimentally [4]. A single administration of psilocybin showed enhanced emotional empathy and divergent thinking. Subacute changes in empathy correlated with changes in well-being [11]. Another group of researchers showed in a study an increase in emotional facial recognition in both healthy individuals and psychiatric patients after receiving psilocybin treatments, giving more validity that it may help in changing cognition that could provide insight into a psychiatric patient’s condition [12].

The amygdala has previously been shown to be involved in the physiology of depression [13]. In addition, psychedelics and many antidepressants have been shown to act on the amygdala. Antidepressants in patients with untreated clinical depression have been shown to attenuate amygdala hypersensitivity [13-15]. Very few neuroimaging studies have been conducted to investigate the effects of psychedelics, thus the effects are largely unknown and poorly understood. In one study, psilocybin was shown through functional magnetic resonance imaging (fMRI) to increase amygdala responses to emotional stimuli. Increased responses to fearful and happy faces were observed in the right amygdala post-treatment with psilocybin, and the right amygdala increases to fearful versus neutral faces were predictive of clinical improvements [16]. In one fMRI study, whole-brain analyses revealed post-treatment decreases in cerebral blood flow (CBF) in the temporal cortex, including the amygdala. Decreased amygdala CBF correlated with reduced depressive symptoms [16,17]. Additional neuroimaging studies need to be conducted to better understand the mechanism and effects of psilocybin.

The abuse potential of psilocybin is very low, with some in fact believing that there is an anti-addictive property to it [18-21]. Some researchers have shown that psilocybin is effective in the treatment of substance use disorders, especially in helping with tobacco cessation in patients who have been unable to quit with other treatment modalities [22,23].

There are numerous methods by which clinicians screen and monitor GAD and depression. For GAD, the most common tools used are the Generalized Anxiety Disorder 7 Item (GAD-7), the Beck Anxiety Inventory (BAI), the Generalized Anxiety Disorder Severity Scale (GADSS), and the State-Trait Anxiety Inventory (STAI) to name a few. For depression, the most commonly used screening and monitoring tools are the Patient Health Questionnaire 9 (PHQ-9), the Beck Depression Inventory (BDI), and the Quick Inventory of Depressive Symptomatology 16-Item Self Report (QIDS-SR16). In the articles for this meta-analysis, many of the above tools were discussed in some capacity. For this meta-analysis, the authors chose to focus on the QIDS-SR16 score across three studies, as this was one measure that had not been used in existing meta-analyses. In a previous meta-analysis from 2020, Vargas et al. used BDI and STAI to observe changes in psychiatric illnesses after the use of psilocybin [24]. Thus, the reasoning for focusing on QIDS-SR16 scores for this second meta-analysis.

Psilocybin treatment has had encouraging results in terms of efficacy, in medium and large doses, especially with adjunctive therapy and careful observation during therapeutic dosing [17,22,23,25,26]. In many studies, the safety and toxicity of psilocybin have been primary goals to show that it is a safe and valid therapeutic alternative to other forms of psychiatric medications. Previous studies have shown promise with dosing 25 mg in a single session. Psilocybin dosing was done with patients who have depression, anxiety, and/or substance use disorder, with most being resistant to at least one other first-line treatment. The current open-label trials have shown a pervasive pattern of individuals not only not needing to take psilocybin everyday, differing from other forms of treatment, but that many patients achieve remission to their condition for a period of time after completion [12,23,25,26]. The need for further research is indicated with more conservative measures and controls and should be attempted to rule out any confounds. Different treatment regimens to determine the best way to apply psilocybin in a clinical setting is the next step suggested in other studies [24,25]. The other potentially positive effect of psilocybin use is indicating what part of the mechanism of action in conjunction with therapy helps create remission. Some researchers have speculated that patients gain “insight” into their condition and are motivated to change their pervasive negative thought patterns as in depression or to make a change in their dependence on a substance as in tobacco abuse [12,22,23].

Studies on psychedelic drugs have shown positive responses in patients with depression, anxiety related to dying, tobacco addiction, and OCD. However, the response to psychedelic drugs is not predictable. While in controlled settings most patients show encouraging responses to psychedelic drugs, in uncontrolled settings, adverse experiences are more frequent. Response to psilocybin is highly correlated to dosage. However, personality traits or mental states at ingestion must be a consideration as adverse experiences have been associated with anxiety, neuroticism, and low trust levels [27]. Sufficient doses of psilocybin evoke mystical, transcendental religious, or spiritual experiences. Insights into God or Ultimate Reality, transcendence of the personal ego, merging with the cosmos, and undergoing transformative death and rebirth are common elements of experiences reported on high doses of psilocybin [22,25]. The importance of mystical experience is that it is a predictor of positive outcomes more than a year after ingestion indicating long-term outcomes [27]. These reports resemble the classical descriptions of self-realization or enlightenment from the mystical traditions of Buddhism and Hinduism. Psychedelics of the past were taken on the principle of mind-expanding or ego-transcending, with doses containing several hundred micrograms. Whereas the current users’ motivations are enhanced sensory experiences during concerts, with the typical dosage being much lower than those in the 1960s, up to 50 mg. The current lighter dose is considered below the threshold for psychedelic revelations [28].

## Review

Methods

The search for this meta-analysis was done through various modalities. PubMed, EBSCOhost, JAMA (Journal of the American Medical Association), and The Lancet were searched in February 2022, and studies were consulted through March 2022. The databases were searched using some of the advanced search features. The articles were limited to those written in English, published within the last 10 years (2012-2022), peer-reviewed, available in free text, and further filtered to only provide results that were randomized controlled trials or clinical trials. Specific phrases or sets of words were also searched: psilocybin and psychiatric illness, psilocybin and PTSD, psilocybin and depression, and psilocybin and psychiatric disorders. A total of nine different searches were conducted, as can be seen in Table 1 below. Reference lists of other articles were also examined to find further potential studies. Each search was given a corresponding search identification number for ease of identifying how each article was obtained.

**Table 1:** Database and article search with corresponding criteria and number of articles. JAMA = Journal of the American Medical Association; PTSD = post-traumatic stress disorder

Search ID	Searches	Search criteria	Number of articles	Database
1	Psilocybin and PTSD	Within the last 10 years, free full-text	16	PubMed
2	Psilocybin and psychiatric illness	Within the last 10 years, free full-text, clinical trials, randomized trials	11	PubMed
3	Psilocybin and depression	Within the last 10 years, free full-text, clinical trials, randomized trials	9	PubMed
4	Psilocybin and psychiatric disorders	Within the last 10 years, free full-text, peer-reviewed, English language	10	EBSCOhost
5	Psilocybin and PTSD	Within the last 10 years, free full-text, peer-reviewed, English language	1	EBSCOhost
6	Psilocybin and depression	Within the last 10 years, free full-text, peer-reviewed, English language	21	EBSCOhost
7	Psilocybin and psychiatric illness	research articles	5	JAMA
8	Psilocybin and psychiatric illness	Research articles in the last 10 years	2	The Lancet
9	Other	Through searching the reference list of previous meta-analyses and/or review articles	3	NA
		Total articles	78	

**Figure 1:**
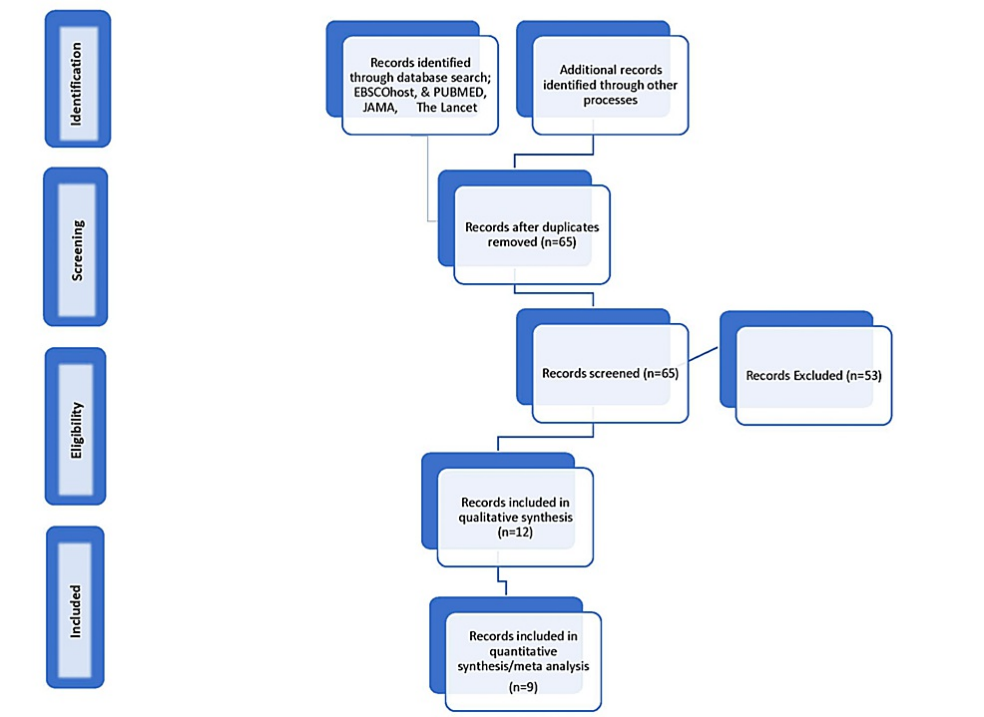
PRISMA flow chart. PRISMA = Preferred Reporting Items for Systematic Reviews and Meta-Analyses; JAMA = Journal of the American Medical Association

A Preferred Reporting Item for Systematic Reviews and Meta-Analyses (PRISMA) flow diagram was created to further aid in the selection process. The selection of articles was independently done by the authors. The PRISMA flow diagram can be seen in Figure 1. Duplicated studies were removed, and some studies were excluded due to duplication of data, as some articles used data from a previous clinical study or randomized controlled trial.

The defined inclusion criteria were studies where the drug psilocybin was used; studies that were randomized controlled trials, clinical trials, or observational studies; studies published from 2012 to 2022; and studies mentioning psychiatric illnesses, including PTSD, anxiety, depression, OCD, or substance abuse.

Searches were done by one author and all authors read, analyzed, and extracted the data. The sample size and the number of participants who showed improvement in psychiatric illness with the use of the drug psilocybin were extracted and data extraction was done independently by the authors. It was decided that data would be used for analysis if the researchers used a method of confirmation that could show the participants’ illness improved from baseline after the study (e.g., biologic confirmation, measures of anxiety, depression, and other similar measures). The length of time participated in treatment, not when the final measures were obtained, was taken into account when assessing proof of improvement. If a participant’s data was unclear or not complete, it was not used in the analysis. Not all measures from each study were used to assess the efficacy of improvement due to the ease of running the meta-analysis and due to inconsistent statistical values used. Each participant who showed improvement on some scale, or had a statistically significant improvement in their illness, was considered a positive event. In the case of no improvement, participants were included as a negative event. Data analysis was used to see if psilocybin caused more positive events than negative events. Extracting data was a challenge due to the different methods used in each study to show a reduction in symptoms/improvement, different treatment methods and lengths, and different illnesses. All psychiatric illness diagnoses were used and some measures may have been subjective, leading the authors to decide with each paper whether the participant improved. This may have limited the validity of our data analysis. A second analysis of data was done using just treatment-resistant depression populations that included a baseline and post-therapy QIDS measurement. This was done as previous meta-analyses had not specifically examined this value across multiple studies.

The extracted data were statistically analyzed using a Microsoft Excel spreadsheet and step-by-step instructions by Neyeloff et al. [29]. Forest and funnel plots were then created using Microsoft Excel to illustrate the data for the original meta-analysis. For the second meta-analysis on QIDS-SR16 scores at baseline and post-treatment with psilocybin, software was downloaded from BioStat Incorporated to analyze the data and create figures [30].

**Figure 2:**
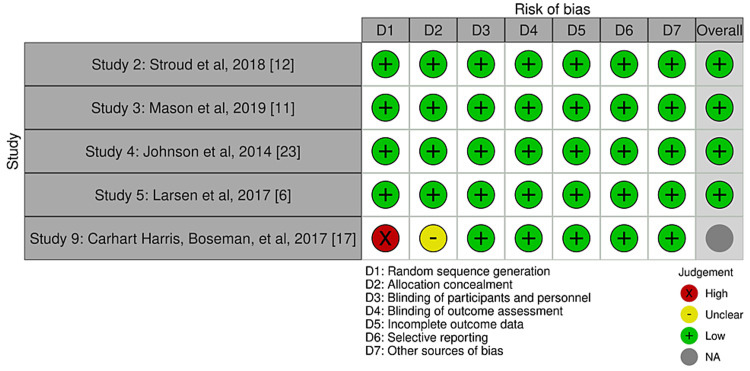
Traffic light plot for randomized controlled trials.

**Figure 3:**
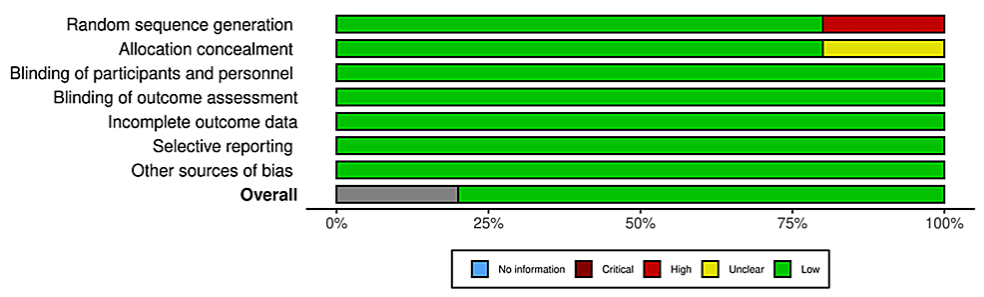
Summary diagram for randomized controlled trials.

**Figure 4:**
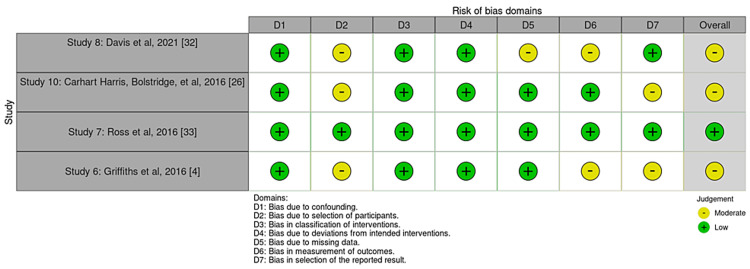
Traffic light plot for non-randomized studies.

**Figure 5:**
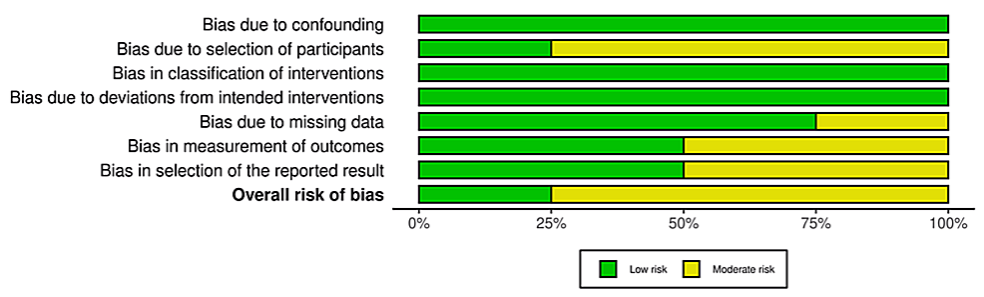
Summary diagram for non-randomized studies.

Bias was reduced using the data from studies that used objective measures to assess progress throughout treatment only. All studies used scales that are highly valid in the psychiatric community to assess a variety of mental health conditions, for example, depression severity, anxiety, etc. Study quality is limited due to many study designs that came without controls or without blinding, as well as many pilot studies. Cochrane bias traffic light plots and summary diagrams can be seen in Figures 2-5. The Risk-of-bias VISualization (ROBVIS) tool by McGuinness was used to generate these diagrams and plots [31]. The generic template was used for randomized controlled trials, and the ROBINS template was used for studies that were not randomized. Cochrane risk of publication bias, or funnel plot, was done as well and can be seen below in the results section.

Results

The PRISMA diagram shows which studies were used in the meta-analysis specifically. Relevant studies that lacked conclusive data were utilized secondarily to contribute to the discussion and provide more background information and supporting statements. Some studies were excluded from the meta-analysis due to duplication of the patient population. While only the final data pool given on the population meeting the inclusion criteria was utilized for the meta-analysis, all studies that were relevant to the target population were cited and discussed.

Nine articles were included in the final data pool, which were used to evaluate the effect of psilocybin on psychiatric illness symptom reduction in patients diagnosed with schizophrenia, PTSD, depression, GAD, and/or substance abuse. The data were grouped together to foster continuity and create a singular outcome, and simplified using parameters of “did this participant do ‘better’ on a valid scale of symptomatic relief” for the pervasive diagnosis. For substance use studies, the question that indicated whether a participant improved was “did the participant abstain from the substance of choice for a period of a six-month follow-up.” In these studies, researchers showed that even if the participant did not abstain, the number of daily uses, of tobacco, for example, was reduced. However, participants’ results were not considered a positive event to keep the criteria rigid.

Post-extraction data are presented in Table 2 and Table 3, showing the sample sizes and events for each study used in the meta-analysis [4,6,11,12,17,23,26,32,33]. The result of treatment after follow-up indicated whether improvement occurred from baseline and this is seen in the events section for each study. Because most studies used the same or similar dosing methods, events were not further divided into different dosing techniques used. In one study, researchers used niacin at baseline and another used a washout period, but again the authors of the present study felt there was no indication to further divide the events for simplicity. Events were also not further divided by different scales as this would complicate analysis due to some studies using up to five scales at baseline and end of treatment. The combined sample size for this meta-analysis was 237. Raw data are presented in Table 4. The results of the risk of publication bias are shown in Figure 3 to indicate the quality of the studies used.

**Table 2:** Theoretical study: improvement in psychiatric illness after use of psilocybin meta-analysis of nine cross-sectional studies/randomized controlled trials. SE = standard error; Var = variance; w = weight; w*es = weighted outcome; w*(es^2^) = weighted outcome squared; w^2^ = weight squared; wv = weighted variance; wv*es = weighted variance outcome; wv*(es^2^) = weighted variance outcome squared; wv^2^ = weighted variance squared

Study	Events	Sample size	Outcome (es)	SE	Var	w	w*es	w*(es^2^)	w^2^	wv	wv*es	wv*(es^2^)	wv^2^
Carhart-Harris et al. 2016 [26]	19	20	0.95	0.22	0.05	21.05	20	19	443.21	7.99	7.60	7.22	63.96
Carhart-Harris et al. 2017 [17]	19	19	1	0.23	0.05	19	19	19	361	7.68	7.68	7.68	59.10
Davis et al. 2021 [32]	17	24	0.71	0.17	0.03	33.88	24	17	1148.01	9.34	6.62	4.69	87.26
Ross et al. 2016 [33]	20.3	29	0.7	0.16	0.02	41.43	29	20.3	1716.32	9.84	6.88	4.82	96.73
Griffiths et al. 2016 [4]	41	51	0.80	0.13	0.02	63.44	51	41	4024.51	10.72	8.62	6.93	114.88
Larsen et al. 2017 [6]	2	12	0.17	0.12	0.01	72	12	2	5184	10.94	1.82	0.30	119.63
Johnson et al. 2014 [23]	12	15	0.8	0.23	0.05	18.75	15	12	351.56	7.64	6.11	4.89	58.39
Mason et al. 2019 [11]	50	50	1	0.14	0.02	50	50	50	2500	10.25	10.25	10.25	105.12
Stroud et al. 2018 [12]	17	17	1	0.24	0.06	17	17	17	289	7.33	7.33	7.33	53.78
Sums						336.55	237	197.3	16017.63	81.74	62.92	54.11	758.78

**Table 3:** Basic statistics for theoretical study: improvement in psychiatric illness after use of psilocybin meta-analysis of nine cross-sectional studies/randomized controlled trials.

Basic statistics	
Weighted sum of squares (Q)	30.40
Weighted sum of squares variance (Qv)	5.68
Degrees of freedom (df)	8
Number of studies in meta-analysis (k)	9
Heterogeneity statistic (I^2^)	73.69
Heterogeneity statistic variance (I^2^v)	-40.84
Outcome, fixed (es (fixed))	0.70
Standard error of outcome, fixed (SEes (fixed))	0.05
Outcome, random (es (random))	0.77
Standard error of outcome, random (SEes (random))	0.11
Confidence interval, fixed (CI (fixed))	0.60, 0.81
Confidence interval, random (CI (random))	0.55, 0.99
Variance (v)	0.08

**Table 4:** Raw data for meta-analysis on improvement in psychiatric illness with the use of psilocybin. SE = standard error; CI = confidence interval; ID = identification

Study	Events	Sample size	Outcome	SE	CI lower	CI upper	Study ID	Rate	CI lower	CI upper
Carhart-Harris et al. 2016 [26]	19	20	0.95	0.22	0.52	1.34	10	95	42.72	42.72
Carhart-Harris et al. 2017 [17]	19	19	1	0.23	0.55	1.45	9	100	44.97	44.97
Davis et al. 2021 [32]	17	24	0.71	0.17	0.37	1.04	8	70.83	33.67	33.67
Ross et al. 2016 [33]	20.3	29	0.7	0.16	0.39	1.00	7	70	30.45	30.45
Griffiths et al. 2016 [4]	41	51	0.80	0.13	0.56	1.05	6	80.39	24.60	24.61
Larsen et al. 2017 [6]	2	12	0.17	0.12	-0.06	0.39	5	16.67	23.10	23.10
Johnson et al. 2014 [23]	12	15	0.8	0.23	0.35	1.25	4	80	45.26	45.26
Mason et al. 2019 [11]	50	50	1	0.14	0.72	1.28	3	100	27.72	27.72
Stroud et al. 2018 [12]	17	17	1	0.24	0.52	1.48	2	100	47.54	47.54
Summary	197.3	237	0.77	0.11	0.55	0.99	1	76.98	21.68	21.68

**Figure 6:**
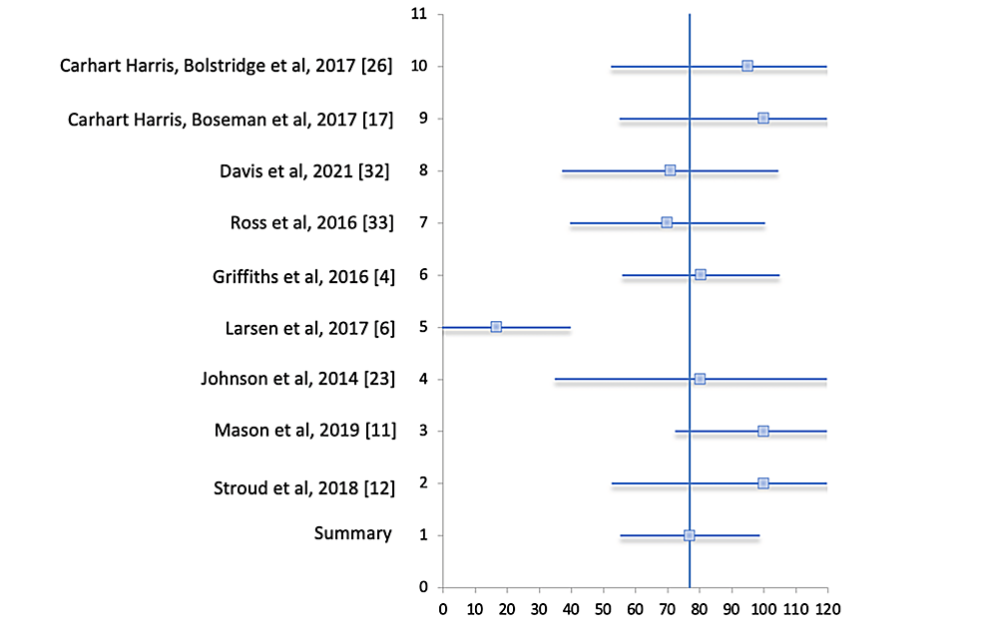
Forest plot for meta-analysis on improvement in psychiatric illness with the use of psilocybin.

The forest plot in Figure 6 below, with corresponding data in Table 5, represents the respective studies along with their confidence intervals. The vertical line represents the line of null effect suggesting that there is no association between an exposure and the outcome or no difference between the two interventions. Each study is represented by a small square. The horizontal lines connected to the blue boxes are the 95% confidence intervals of the study result. The end of each line represents the boundaries of the 95% confidence interval. If the horizontal lines cross the line of null effect, this would indicate that the null value lies within the confidence interval and could be the true value. The studies with a greater number of participants will show a more narrow confidence interval and therefore a smaller horizontal line. In the forest plot in Figure 6, one can see that lines 1-4 and 6-10 cross the line of null effect. Line 1, indicated by the diamond-shaped plot, represents the summary of all studies, horizontal line 2 represents the study by Stroud et al., horizontal line 3 represents the study by Mason et al., and so on, as shown in the corresponding table below the forest plot.

**Table 5:** Forest plot data for meta-analysis on improvement in psychiatric illness with the use of psilocybin. CI = confidence interval

Study	Rate (95% CI)
Carhart-Harris et al. 2016 [26]	95 (52.3-137.7)
Carhart-Harris et al. 2017 [17]	100 (55.0-145)
Davis et al. 2021 [32]	70.83 (37.2-104.5)
Ross et al. 2016 [33]	70 ( 39.5-100.5)
Griffiths et al. 2016 [4]	80.39 (55.8-105.0)
Larsen et al. 2017 [6]	16.67 (-6.4-39.8)
Johnson et al. 2014 [23]	80 (34.7-125.3)
Mason et al. 2019 [11]	100 (72.3-127.7)
Stroud et al. 2018 [12]	100 (52.5-147.5)
Summary	76.98 (55.3-98.7)

The I^2^ statistic can be seen in Table 3, with the value being 73.68%. The I^2^ value indicates that the studies are inconsistent, likely due to a reason other than chance. Ideally, one would like to see the I^2^ statistic less than 50%. The I^2^ statistic is the percentage of the total variability in a set of effect sizes due to true heterogeneity between studied variability [29]. A high I^2^ value suggests that the studies in this meta-analysis cannot be considered to be studies of the same population [34].

The Q value, or Cochrane’s Q, is 30.4 which is higher than 15.507, the critical value for eight degrees of freedom found in a chi-square distribution. Cochrane’s Q represents the weighted sum of squared differences between the observed effects and the weighted average effect [33]. Cochrane’s Q is a measure of variation around the mean and is not a measure of heterogeneity.

**Figure 7:**
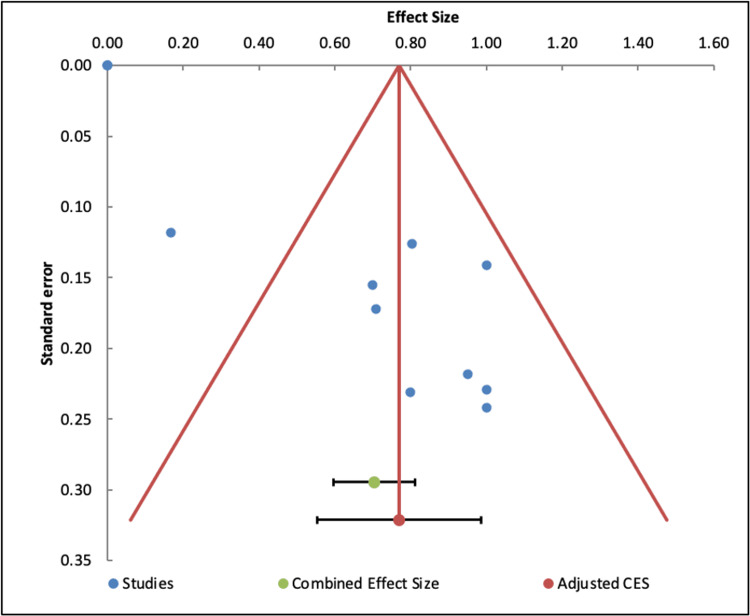
Funnel plot showing potential publication bias in the meta-analysis on improvement in psychiatric illness with the use of psilocybin.

The results of this funnel plot, seen in Figure 7, cannot be interpreted due to the high degree of heterogeneity in the set of effect sizes based on the I^2^ value. Usually, publication bias analyses, represented in funnel plots, are only performed in a set of homogenous results. It is assumed that observed effect sizes with similar precision, for example with similar standard error, should be more or less symmetrically distributed around the combined effect size [34].

As shown in Table 6 and Table 7 below, an additional meta-analysis was done on three prior studies that reported changes in the QIDS-SR16 score at baseline and again after treatment with psilocybin. As seen in Table 6, the mean of the QIDS-SR16 score at both baseline and after treatment with psilocybin was used, in addition to the standard deviation, and the sample size. A total combined sample size of 60 was used for this second meta-analysis. Table 7 demonstrates fixed-effects statistics. Table 8 demonstrates the Q value and the I^2^ statistic. The Q value for this second meta-analysis is 1.16 which is lower than 5.991, the critical value for two degrees of freedom found in a chi-square distribution. The I^2 ^statistic for the second meta-analysis is -73% which can be equated to zero. This indicates that there is no observed heterogeneity in the data. Due to low heterogeneity, the fixed-effects model was used. The p-value seen in Table 7 from this second meta-analysis is less than 0.05, indicating that this meta-analysis is statistically significant.

**Table 6:** Meta-Analysis of QIDS-SR16 scores across three studies. N = number of participants; D = raw difference in means; d = standardized difference in means or Cohen’s d; g = standardized difference in means or Hedgen’s d; V_d_ = variance of d; V_g_ = variance of g; Y = effect size; V_Y_ = variance within; W = weight; WY = calculated quantity weight of effect size; WY^2^ = calculated quantity weight of effect size squared; W^2^ = weight squared; QIDS-SR16 = Quick Inventory of Depressive Symptomatology 16-Item Self Report

Study	Treated			Control/Baseline										Effect size	Variance within	Weight	Calculated quantities		
	Mean of QIDS-SR16 Score	SD of QIDS-SR16 Score	N	Mean of QIDS-SR16 Score	SD of QIDS-SR16 Score	N	D	S_within_	d	V_d_	J	g	V_g_	Y	V_Y_	W	WY	WY^2^	W^2^
	Stroud et al. 2018 [12]	7.65	5.34	17	18.88	2.23	17	-11.23	4.09	-2.744	0.23	0.98	-2.68	0.22	-2.68	0.22	4.59	-12.30	32.98	21.09
Carhart-Harris et al. 2017 [17]	8.8	6.2	19	18.9	3	19	-10.1	4.87	-2.07	0.16	0.98	-2.03	0.16	-2.03	0.16	6.45	-13.09	26.57	41.56
Davis et al. 2021 [32]	6	5.7	24	16.7	3.5	24	-10.7	4.73	-2.26	0.14	0.98	-2.225	0.13	-2.23	0.13	7.56	-16.83	37.46	57.22

**Table 7:** Fixed-effects statistics for meta-analysis of QIDS-SR16 scores across three studies. QIDS-SR16 = Quick Inventory of Depressive Symptomatology 16-Item Self Report

Mean and precision	
Mean effect (M)	-2.27
Variance of M (VM)	0.05
Standard error of mean (SeM)	0.23
Confidence intervals	
Lower limit (95%) (LLM)	-2.72
Upper limit (95%) (ULM)	-1.82
Test of the null that M = 0	
Z for test of null (Z)	-9.79
P-value (one-tailed) (p1)	0.00
P-value (two-tailed) (p2)	0.00

**Table 8:** Heterogeneity statistics for meta-analysis of QIDS-SR16 scores across three studies. QIDS-SR16 = Quick Inventory of Depressive Symptomatology 16-Item Self Report

Heterogeneity statistics	
Basic statistics	
Weighted sum of squares (Q)	1.16
Degrees of freedom (df)	2
P-value for Q (p(Q))	0.56
Scaling factor (C)	12.16
Scaling factor (A)	2
T2 and related statistics	
Between-studies variance (T2)	0
Variance of T2 (VT2)	0.03
Standard error of T2 (SET2)	0.16
Intermediate value B (B)	1.30
Intermediate value L (L)	0.06
Intermediate value U (U)	9.64
Lower limit of T2 (95%) (LLT2)	0
Upper limit of T2 (95%) (ULT2)	15.11
Tau and related statistics	
Between-studies standard deviation (T)	0
Lower limit of T (95%) (LLT)	0
Upper limit of T(95%) (ULT)	3.89
I^2 ^and related statistics	
I^2^	-73.01%
Lower limit of I^2^ (95%) (LLI2)	0.00%
Upper limit of I^2^ (95%) (ULI2)	98.92%
Prediction intervals	
Degrees of freedom (df)	1
Critical value for t (95% interval) (tCrit)	12.70
Mean effect (random effect weights) (M*)	-2.27
Tau-squared (T2)	0
Variance of M* (VM*)	0.05
Prediction interval (95%) lower limit (LLPred)	-5.21
Prediction interval (95%) upper limit (ULPred)	0.68

**Figure 8:**
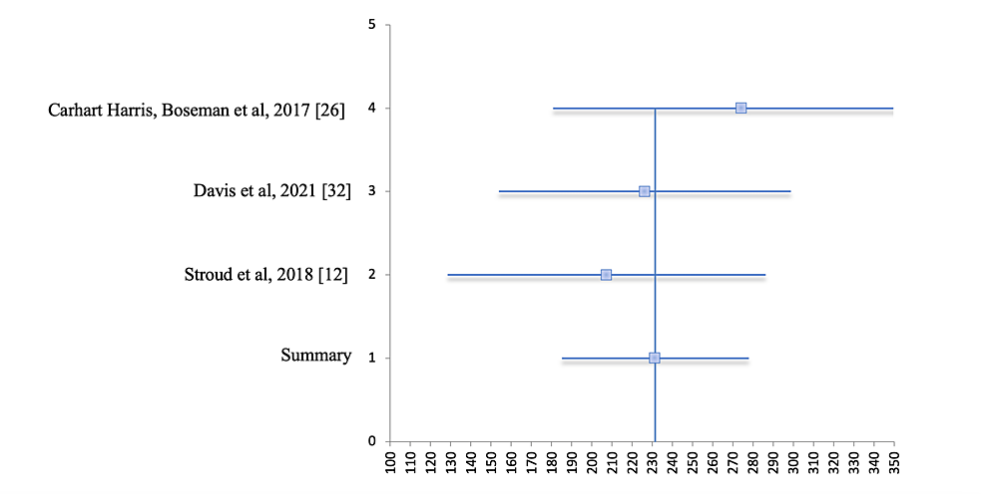
Forest plot for meta-analysis of QIDS-SR16 scores across three studies. QIDS-SR16 = Quick Inventory of Depressive Symptomatology 16-Item Self Report

**Figure 9:**
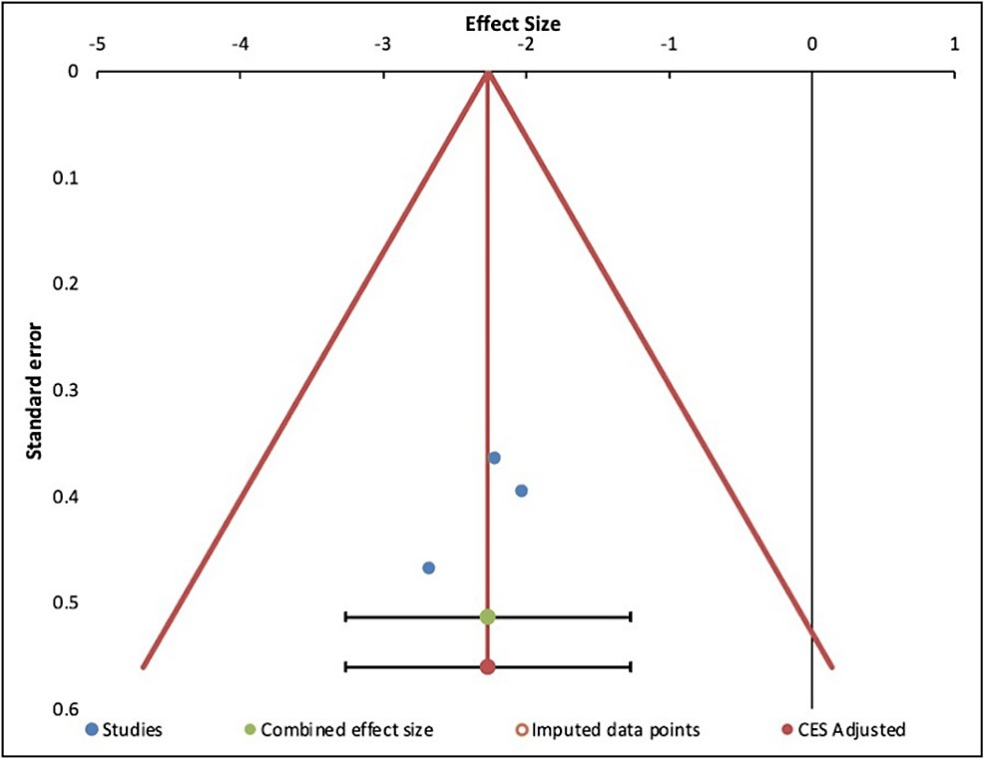
Funnel plot for meta-analysis of QIDS-SR16 scores across three studies. QIDS-SR16 = Quick Inventory of Depressive Symptomatology 16-Item Self Report

Figures 8 and 9 show the forest plot and funnel plot, respectively, for the second meta-analysis that was done on three studies for the QIDS-SR16 scores. This demonstrates that there is not likely to be publication bias. In the forest plot in Figure 8, lines 1-4 cross the line of null effect. Line 1 represents the summary of all studies, horizontal line 2 represents the study by Stroud et al., horizontal line 3 represents the study by Davis et al., and so on, as shown in the corresponding table below the forest plot (Table 9). In the funnel plot below in Figure 9, one can see that it is symmetrical, with all studies being grouped closely around the combined effect size.

**Table 9:** Forest plot raw data for meta-analysis of QIDS-SR16 scores across three studies. SE = standard error; CI = confidence interval; QIDS-SR16 = Quick Inventory of Depressive Symptomatology 16-Item Self Report

Study	Events	Sample Size	Outcome	SE	CI lower	CI upper	Study number	Rate	CI lower	CI upper
Carhart-Harris et al. 2017 [26]	19	19	2.74	0.48	1.81	3.68	4	274.4	93.6	93.7
Davis et al. 2021 [32]	17	24	2.26	0.37	1.54	2.99	3	226.2	72.4	72.5
Stroud et al. 2018 [12]	17	17	2.07	0.40	1.29	2.86	2	207.4	78.9	78.8
Summary			2.31	0.24	1.85	2.78	1	231.5	46.3	46.4

Discussion

Microdosing of psilocybin has been shown to be safe and efficacious in recent open-label and randomized controlled trials. The indications for psilocybin as a future treatment modality are yet to be fully determined. Its value comes as an adjunctive treatment to psychotherapy and primary treatment for resistant cases, as well as to help decrease the relapse potential of a psychiatric condition when used for several sessions. Further, when psilocybin is used in a closed environment under close observation, the side effect profile of headache and occasional nausea is limited to the euphoric and hallucinogenic session itself for several hours to days after sessions; there is no indication of long-term side effects when used for only a few sessions [17,25,26]. This differs greatly from treatment with some neuroleptic medications and selective serotonin reuptake inhibitors (SSRIs) commonly used for psychiatric illnesses.

In comparison to other treatments such as ketamine, where an effect was only seen to last a few days to two weeks, psilocybin was shown to have effects lasting from four to eight weeks, with a ≥50% reduction in the GRID-Hamilton Depression Rating Scale (GRID-HAMD) score with only two sessions of treatment. Along with the higher efficacy, it was also noted that psilocybin participants experienced lower rates of dependency or addiction when compared to study participants using ketamine or other pharmaceuticals, which prompted further usage [34]. Aside from having reductions in the GRID-HAMD scores, studies also revealed a significant decrease when tested with the QIDS-SR16 as well with all patients in a study showing a drastic decrease in their scores with the changes being present up to five weeks post-treatment. A second meta-analysis was conducted in this study in which three of the nine studies used the QIDS-SR16 as a measure and showed a decrease in the scores. These studies were analyzed and the QIDS-SR16 scores were compared across the board. Cochrane’s Q showed that the data was homogenous, and a p-value of less than 0.05 was calculated, indicating that the results of the meta-analysis were statistically and clinically significant.

In addition to depression scaling, it was noted that psilocybin had an effect on overall cerebral blood flow, especially showing a decrease in the blood flow to the amygdala within just one-day post-treatment. The decrease in blood flow to the amygdala correlated with the decrease in depressive symptoms, supporting the efficacy of the usage of psilocybin treatment [17].

Along with decreased adverse effects and decreased dependency on psilocybin, the tremendous and drastic effects of the treatment seen through minimal usage are also positive outcomes when attempting to manage psychiatric illnesses in comparison with psychotherapy and/or other pharmaceuticals. In many of the studies, researchers administered psilocybin by microdosing, allowing responses to be seen at the cellular level instead of whole-body effects. When participants were treated with two treatment sessions one week apart with doses of 20 mg for the first treatment and 30 mg for the second treatment, the effects were noticeably present six months after treatment completion [23,26]. In comparison, SSRIs or other pharmaceuticals consisted of daily treatment, often with minimal effectiveness, and in some cases with symptoms resisting treatment [34].

The results of the present study remain unclear due to a large degree of heterogeneity, after analyzing the data in the original meta-analysis where nine studies were used, making it difficult to endorse the prescription of psilocybin in the treatment of psychiatric conditions. Because the analysis resulted in a high degree of heterogeneity, it is unclear if the results are generalizable to the general population. Analysis of the QIDS-SR16 scores in the treatment-resistant population revealed a statistically significant effect, warranting continued research to determine appropriate applications for psilocybin, and to compare it to the effectiveness of the current gold-standard therapies. We can unequivocally say that there is a statistically significant difference in the improvement of psychiatric conditions with the use of psilocybin, from baseline in the populations studied, and it should be used to further understanding. Recommendations for future research include using controls with the gold-standard treatments as well as having several treatment groups at different doses and dosing schedules. Studying participants with varying levels of symptom severity followed for a longer period of time would further the search for safe and effective treatment alternatives.

The strengths of this meta-analysis consist of involving most studies that were conducted under randomized design and some studies requiring that participants discontinue any psychiatric pharmaceuticals prior to the start of psilocybin treatment. As research shows, serotonergic antidepressants downregulate the 5-HT2A receptor which is the primary receptor target of psilocybin. Ensuring that patients withdraw from taking any antidepressant medication prior to initiating psilocybin treatment allowed for the results of the psilocybin treatment alone to be more efficacious and supportive of its use [26].

There are numerous limitations to this meta-analysis. Pharmaceutical companies cannot patent the drug so less money is invested into research by government entities, thus the need for increased private funding and further research on the topic. Many studies involved in this meta-analysis do not use or discuss the amount of time or money that goes into these treatments which may affect whether psilocybin is a cost-effective and time-sensitive treatment. The tobacco use patients were able to stay tobacco-free for six months or longer, and better outcomes in depression and anxiety may be a confound. The possibility of greater access to high-quality and longer treatment time, for example, spending more hours with the therapist, and superior care may have affected the outcome of these studies. When compared to what is possible in the clinical trial setting, these studies may not be generalized to psychiatry as a field. In addition, many studies used psilocybin in addition to psychotherapy, thus it is unclear if the improvements are due to psilocybin alone or if they are due to the combination of psilocybin and psychotherapy. Other limitations among the studies consist of short-term follow-up with small sample sizes. Having long-term follow-ups with larger sample sizes would allow us to confirm the long-term efficacy among a more diverse population. In addition, the use of articles that were only available via free text could be another limitation of this study. There are numerous ongoing studies and clinical trials that could change the landscape regarding psychedelics as a therapy for psychiatric illnesses and positively change the lives of countless individuals.

## Conclusions

Psilocybin has been used in clinical research to treat a number of psychiatric illnesses with varying degrees of effectiveness. Because psilocybin is a schedule I substance under the Controlled Substances Act, there is a limited number of randomized control trials or clinical trials. The current understanding in academia is that psilocybin is safe to use in a controlled setting, and patients ingesting microdoses have shown symptom improvement with some psychiatric illnesses. The effectiveness of psilocybin microdosing and the use of psilocybin, in general, need to be studied further to determine the efficacy and safety of potential applications in psychiatry.

This is especially true if some of the results of our analysis can be replicated in the outside population consistently. The effects that these trials have had on individuals have been encouraging. However, further research is needed to determine if these effects are lasting. The hope for forms of treatment involving microdosing is that individuals develop lasting beneficial effects from short-term treatment. Although microdosing psychedelic treatment may always carry with it a stigma, studies such as this one as well as future studies will hopefully help solidify the concept as a legitimate option as a form of treatment for a myriad of mental issues.
